# Oxygen dependency of mitochondrial metabolism indicates outcome of newborn brain injury

**DOI:** 10.1177/0271678X18777928

**Published:** 2018-05-18

**Authors:** Gemma Bale, Subhabrata Mitra, Isabel de Roever, Magdalena Sokolska, David Price, Alan Bainbridge, Roxana Gunny, Cristina Uria-Avellanal, Giles S Kendall, Judith Meek, Nicola J Robertson, Ilias Tachtsidis

**Affiliations:** 1 Department of Medical Physics and Biomedical Engineering, University College London, London, UK; 2 Institute of Women's Health, University College London, London, UK; 3 Department of Medical Physics and Biomedical Engineering, University College London Hospital, London, UK; 4 Paediatric Neuroradiology, Great Ormond Street Hospital for Children, London, UK; 5 Neonatal Unit, University College London Hospital, London, UK

**Keywords:** Cerebral haemodynamics, metabolism, mitochondria, near-infrared spectroscopy, perinatal hypoxia

## Abstract

There is a need for a method of real-time assessment of brain metabolism during neonatal hypoxic-ischaemic encephalopathy (HIE). We have used broadband near-infrared spectroscopy (NIRS) to monitor cerebral oxygenation and metabolic changes in 50 neonates with HIE undergoing therapeutic hypothermia treatment. In 24 neonates, 54 episodes of spontaneous decreases in peripheral oxygen saturation (desaturations) were recorded between 6 and 81 h after birth. We observed differences in the cerebral metabolic responses to these episodes that were related to the predicted outcome of the injury, as determined by subsequent magnetic resonance spectroscopy derived lactate/N-acetyl-aspartate. We demonstrated that a strong relationship between cerebral metabolism (broadband NIRS-measured cytochrome-c-oxidase (CCO)) and cerebral oxygenation was associated with unfavourable outcome; this is likely to be due to a lower cerebral metabolic rate and mitochondrial dysfunction in severe encephalopathy. Specifically, a decrease in the brain tissue oxidation state of CCO greater than 0.06 µM per 1 µM brain haemoglobin oxygenation drop was able to predict the outcome with 64% sensitivity and 79% specificity (receiver operating characteristic area under the curve = 0.73). With further work on the implementation of this methodology, broadband NIRS has the potential to provide an early, cotside, non-invasive, clinically relevant metabolic marker of perinatal hypoxic-ischaemic injury.

## Introduction

Hypoxic-ischaemic encephalopathy (HIE) is responsible for a quarter of neonatal deaths globally^1^ and is the second most common cause of preventable childhood disability.^2^ In developed countries, the incidence of HIE is around 1.5 per 1000 live births, with 5- to 10-fold higher rates in mid and low resource settings.^3^

Following resuscitation after perinatal hypoxia-ischaemia (HI), the neonatal brain evolves through a period of partial recovery, followed by a latent phase (the probable therapeutic window).^4^ Following this, a secondary phase of energy failure may occur which is associated with cytotoxic oedema, cell death due to mitochondrial injury and clinical deterioration often with seizures.^5^ Therapeutic hypothermia (HT) started during the latent phase reduces secondary energy failure, cell death and improves outcome at 18–24 months and at school age.^6–8^ Although HT has been standard care for babies with moderate to severe HIE, around 50% of treated infants have adverse outcomes. Research into adjunct neuroprotective therapies is currently an area of focus,^4,9^ so a continuous monitor of brain tissue health is highly desirable.

The progression of the brain health during HIE can be characterised in terms of the metabolism. The metabolic changes during and after a hypoxic-ischaemic insult in an animal model have been measured using proton (^1^H) magnetic resonance spectroscopy (MRS).^10^ An increase in lactic acid occurs during hypoxia-ischaemia (produced as a result of anaerobic respiration). Following resuscitation and return of oxygen and substrate supply, aerobic respiration resumes and the brain lactate (Lac) returns to almost normal levels during the latent phase. However, a secondary rise in brain lactate and reduction in *N*-acetyl-aspartate (NAA), suggesting mitochondrial dysfunction and loss of neuronal integrity, occurs during the secondary energy failure due to an evolving cascade of injury.^10^ The ^1^H MRS derived thalamic Lac/NAA peak area ratio is a robust predictor of neurodevelopmental outcome in babies with HIE^11,12^ and has been used as surrogate outcome measures in clinical neuroprotection studies of HIE.^13^ This metabolic information is vital for prognostication and counselling, but only gives a snapshot of the cerebral injury at a particular time point, usually after subacute secondary energy failure phase. Identification of worsening brain injury and potential unfavourable outcome with a bedside tool during treatment could improve outcome of these patients.

### Broadband near-infrared spectroscopy

Near-infrared spectroscopy (NIRS) can yield information about cerebral haemodynamics (via oxy- and deoxy-haemoglobin: HbO_2_ and HHb, respectively) and tissue oxygenation at the cotside. The use of NIRS systems to monitor the haemodynamics of HIE has been demonstrated by many research teams over the past 20 years,^14–25^ yet none have identified a difference in the levels of injury. Measurement of cerebral metabolism, which is related to neuronal activity, is likely to be more sensitive to different severities of injury.^17,18,21,26,27^

Broadband NIRS additionally monitors changes in the activity of cytochrome-c-oxidase (CCO) and has the potential to provide a metabolic marker. CCO is the terminal electron acceptor in the electron transport chain (ETC): the final stage of oxidative metabolism.^26^ A unique copper dimer (Copper A) in the enzyme has an absorption peak around 835 nm in its oxidised form (oxCCO), but not in its reduced state. A change in the redox state represents a change in oxidative cellular metabolism. To accurately resolve changes in oxCCO, many wavelengths (broadband) are required as the concentration of CCO is 10% of the in vivo haemoglobin concentration.^28,29^ Broadband NIRS-measured oxCCO changes are associated with acute changes in metabolism following hypoxia-ischaemia;^26,30–33^ see Bale et al.^28^ for a detailed review.

### Aim

Our aim was to determine whether broadband NIRS can distinguish injury severity in HIE in the first 4 days after birth. It has been established that a disruption of the cerebral metabolic rate is associated with severe brain injury.^17,18,20,21,26^ Probing this relationship further, we assessed the metabolic and haemodynamic responses to spontaneous episodes of hypoxia (desaturations) in newborns with favourable and unfavourable outcome after HIE. We hypothesised that the relationship between cerebral tissue oxygenation and mitochondrial function would indicate the severity of the injury; whereby in brain injury resulting in an unfavourable outcome, mitochondrial function is more dependent on oxygenation.

## Materials and methods

### Study participants and protocol

This prospective observational study (Baby Brain Study) was approved by the Research Ethics Committee (REC) of University College London Hospital and London Bloomsbury REC (reference: 13/LO/0106) in accordance with the Helsinki Declaration. Written, informed consent was obtained from parents before each study. Parents of term infants born at or transferred to UCLH for treatment of HIE were approached for informed consent; we excluded babies with congenital malformations. As per the national guideline (NICE Guidelines) infants determined to have moderate to severe HIE were cooled to 33.5℃ for 72 h as soon as possible after birth. Rewarming was started after 72 h, increasing 0.5℃ every 2 h over a period of 14 h. All infants were intubated and ventilated during HT, and received continuous morphine and atracurium infusions, as is standard policy in the UCLH NICU. Data were collected from 50 infants with HIE over the first 4 days of life during HT and rewarming; specifically, the neonates were monitored from as early as 5 h until 96 h after birth. The total duration of the monitoring varied between infants and ranged from 8 to 78 h (see Table 1).

**Table 1.** Details of neonates studied with desaturation events eligible for analysis.

Neonate	Gender	GA (weeks)	Birth weight (g)	Lac/NAA	Outcome prediction	Duration (hours)	No. of events	Time of events (hours from birth)
2	F	38.0	1770	0.87	Unfavourable	29.0	1	37
3	F	41.0	3800	0.2	Favourable	47.1	9	23, 71, 71, 71, 72, 72, 75, 76, 78
6	M	40.3	3110	0.39	Unfavourable	29.1	2	56, 62
7	M	39.7	3640	1.32	Unfavourable	24.7	2	67, 68
8	F	41.9	3498	0.17	Favourable	26.8	1	51
9	M	38.9	2850	0.16	Favourable	35.3	5	34, 35, 36
11	F	40.6	3580	0.08	Favourable	77.5	1	37
14	M	37.7	3750	0.25	Favourable	30.9	2	14, 54
15	F	39.6	3240	0.15	Favourable	36.4	3	16, 70, 78
17	F	37.4	3160	0.35	Unfavourable	50.2	2	13, 37
19	F	39.4	3700	0.16	Favourable	28.9	1	18
20	M	40.9	3190	0.11	Favourable	48.7	2	40, 66
21	M	36.4	2440	0.2	Favourable	41.1	3	33, 41, 81
25	M	41.9	4940	0.4	Unfavourable	8.1	1	78
35	F	38.7	3150	0.14	Favourable	12.0	1	18
37	F	39.4	3330	0.2	Favourable	10.1	2	12, 14
47	F	41.7	3390	0.41	Unfavourable	16.9	1	51
48	M	39.0	3340	2.64	Unfavourable	29.0	1	69
51	M	38.1	3070	0.18	Favourable	15.6	2	58, 58
53	F	37.6	3020	0.17	Favourable	16.9	2	17, 34
56	M	40	3365	0.34	Unfavourable	13.5	1	29
59	M	41.1	3990	0.16	Favourable	15.5	3	17, 43, 44
60	F	40.7	2754	0.15	Favourable	15.7	3	6, 7, 9
62	F	40	3442	0.21	Favourable	25.8	3	30, 53, 58

Continuous systemic data from bedside Intellivue Monitors (Philips Healthcare, UK) were collected simultaneously with the NIRS data using an application called ixTrend (ixellence GmbH, Germany). Signals recorded include oxygen saturation (SpO_2_), heart rate, respiratory rate and mean arterial blood pressure.

### Magnetic resonance imaging and spectroscopy

MRI scans with ^1^H MRS were performed between days 5 and 10 using a 3T Philips MRI scanner (Philips Healthcare) and processed with jMRUI (v4). T1 and T2 weighted imaging sequences were obtained along with diffusion-weighted imaging sequences. A single voxel of 1.5 cm × 1.5 cm × 1.5 cm was positioned to encompass as much of left thalamus as possible whilst avoiding overlap with CSF to obtain MRS spectra. Thalamic Lac/N-acetyl aspartate peak area ratio obtained from ^1^H MRS is the most accurate quantitative MR biomarker within the neonatal period for prediction of neurodevelopmental outcome following HIE;^11^ Lac/NAA < 0.3 was noted to indicate good outcome in this meta-analysis.

### Broadband NIRS instrumentation and processing

**Figure 1.**
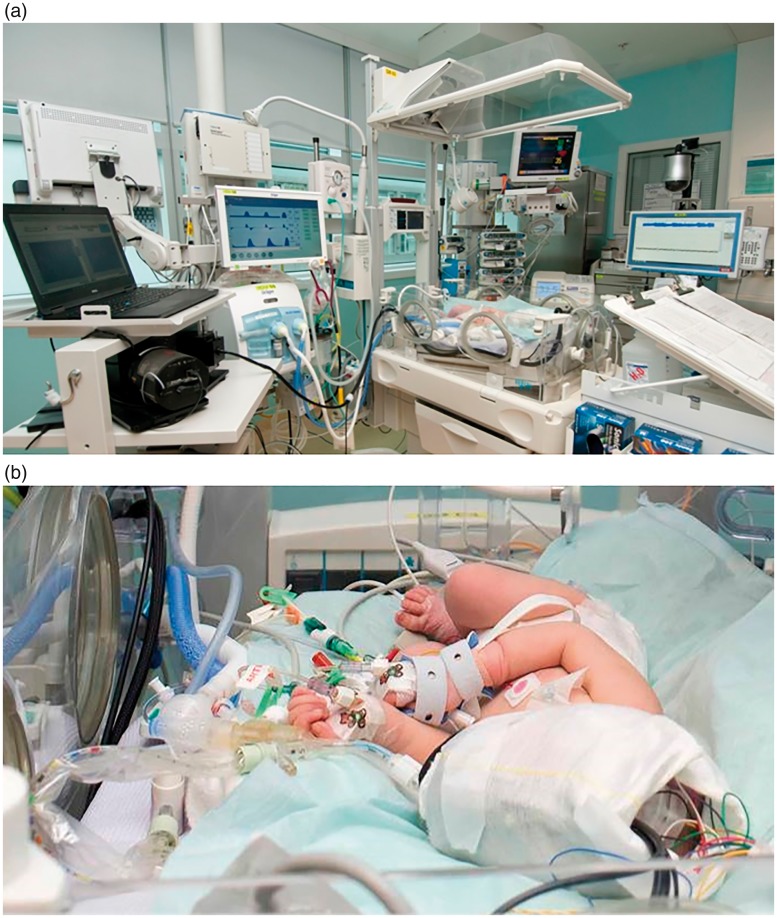
(a) Experimental set up demonstrating the integration of the broadband NIRS system within the multimodal monitoring environment in the NICU: broadband NIRS system is on the left with optical fibres (black) entering the cot. (b) The baby in the cot being monitored during treatment for HIE with broadband NIRS (black optical fibre cables), EEG, transcutaneous monitors, blood pressure catheter, respirator and ECG.

The broadband NIRS device used in this study has been previously described.^34^ Briefly, the system consists of an optical fibre illuminator (ORIEL 77501, Newport, UK) and a lens-based spectrometer (LS785, Princeton Instruments) with a front-illuminated CCD camera (PIXIS 512f, Princeton Instruments) (see Figure 1(a)). An optical fibre bundle carries light to the tissue, and another detector bundle collects the attenuated light emerging from the tissue; the fibres are held onto the head with a 3D printed fibre holder. All measurements were taken on the forehead over the right hemisphere of the frontal lobe with a source-detector separation of 30 mm (see Figure 1(b)).

Detected intensity data were collected at 1 Hz using custom-built software (LabVIEW 2011, National Instruments). Attenuation changes across the 770–905 nm wavelength range were used to resolve concentration changes in HbO_2_, HHb and oxCCO using the UCLn algorithm.^28,35^ A constant pathlength was assumed with a differential pathlength factor of 4.99^36^ which was corrected for the wavelength dependency of the pathlength.^37^

### Data analysis

All data analysis was done in MATLAB 2013b (Mathworks). Automatic synchronisation of the broadband NIRS data with the systemic data was performed.

Data from during the therapeutic hypothermia period was included in the analysis; that is events occurring on postnatal days 1–3 during hypothermia (33.5℃) and day 4 during the rewarming period but still at hypothermic temperatures (<35℃).

**Figure 2.**
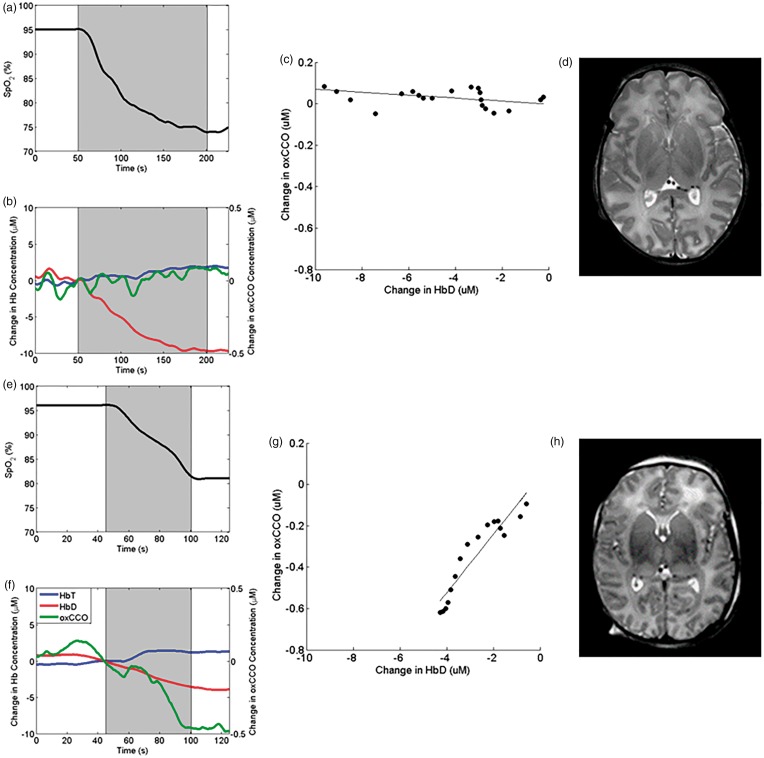
Examples of broadband NIRS and MR data from two infants with different HI injury severities. (a–d) Neonate 021 with favourable outcome: (a) pulse oximeter recording of SpO_2_ during desaturation event (grey). (b) NIRS recording of HbD, HbT and oxCCO during same desaturation event (grey) as in (a). Note that oxCCO is plotted on the right axis. (c) Change in oxCCO against HbD during desaturation period from (a) and (b) showing a gradient of −0.01. (d) T2 weighted MRI on day 7 revealed high signal intensity in the white matter (WM) with normal deep grey matter (DGM). Lac/NAA was 0.2. (e–h) Neonate 007 with unfavourable outcome. (e) Pulse oximeter recording of SpO_2_ during desaturation event (grey). (f) NIRS recording of HbD, HbT and oxCCO during same desaturation event (grey) as in (e). Note that oxCCO is plotted on the right axis. (g) Change in oxCCO against HbD during desaturation period from (e) and (f) showing a gradient of 0.14. (h) T2 weighted MRI on day 5 revealed widespread signal intensities both in WM and DGM. Lac/NAA was 1.32.

Multimodal data were examined during clinically stable periods (no seizure activity), and desaturation events were selected. A drop in SpO_2_ to below 85% from above 95% was selected as a hypoxic event (see examples in Figure 2(a) and (e)). If there were no hypoxic events identified in their data, the neonate was excluded from this analysis. In cases where there were multiple events per baby, the events were averaged before the group analysis. After event selection, NIRS data were filtered using a third order Savitzky–Golay filter and normalised to 0 µM at the event start time. As each event has a different duration and SpO_2_ decrease, the NIRS and systemic data were averaged per each 5% change in SpO_2_.

^1^H MRS derived thalamic Lac/NAA was used as a clinical biomarker as a prognostic measure of outcome.^11^ Favourable outcome was predicted by Lac/NAA < 0.3. It was not possible to observe the severity of the original injury because a significant proportion of the neonates were transferred ex utero from different local units for management of HIE and were already on medication on arrival at our NICU. The median and interquartile range of the signals was calculated for each NIRS and systemic signal at each 5% SpO_2_ for each group (minimum of four events).

### Statistical analysis

The significance of the difference between the group NIRS variables and baseline was assessed using Kruskal–Wallis tests (*p* < 0.05 was considered statistically significant).

The gradients of oxCCO against the HbD, HbT and SpO_2_ changes during each desaturation were found for each event, and receiver operating characteristic (ROC) curves were used to illustrate the performance of the gradients of the events as classifiers of injury.

## Results

A total of 54 arterial desaturation events were recorded in 24 neonates (8 unfavourable and 16 favourable outcomes after HIE, as determined by MRS-measured Lac/NAA) out of 50 neonates monitored with broadband NIRS; data from 26 neonates were excluded. All events were recorded over postnatal days 1–4 during hypothermia: the first event was recorded 6 h after birth, and the last event was at 81 h. Table 1 shows the clinical details of the infants included in this analysis, and the number and timing of events recorded per infant (see online Supplementary Table 1 for clinical details for all infants studied, including those without desaturation events eligible for analysis). Table 2 shows the mean changes in the systemic parameters measured during the desaturation event periods. The average desaturation period occurred over 131 ± 97 s, with a range from 13 to 455 s. The average SpO_2_ nadir was 65% ± 21%, with a range from 8% to 85%. The mean birth weight was 3.3 ± 0.6 kg, and the mean GA was 39.6 ± 1.5 weeks; 13 neonates were female. A total of 54 events were identified: 11 from neonates with unfavourable outcome (predicted by MRS measured Lac/NAA > 0.3) and 43 from neonates with a favourable outcome.

**Table 2.** Mean ± standard deviation of the systemic parameters during baseline and nadir of desaturation events.

	Baseline		Nadir	
Outcome	Favourable	Unfavourable	Favourable	Unfavourable
SpO_2_ (%)	95.94 ± 2.20	97.35 ± 2.03	66.74 ± 21.92	78.17 ± 5.35
MABP (mm Hg)	52.27 ± 10.09	50.25 ± 9.01	50.25 ± 9.78	50.54 ± 8.03
HR (bpm)	114.23 ± 18.03*	130.88 ± 18.47*	115.29 ± 18.88*	130.54 ± 18.86*
RR (r/min)	29.83 ± 10.31	25.04 ± 12.19	31.37 ± 9.79	34.06 ± 18.75

Note: Asterisks show a significant difference between the groups (*p* < 0.05). SpO_2_: oxygen saturation; MABP: mean arterial blood pressure, HR: heart rate, RR: respiratory rate.

**Figure 3.**
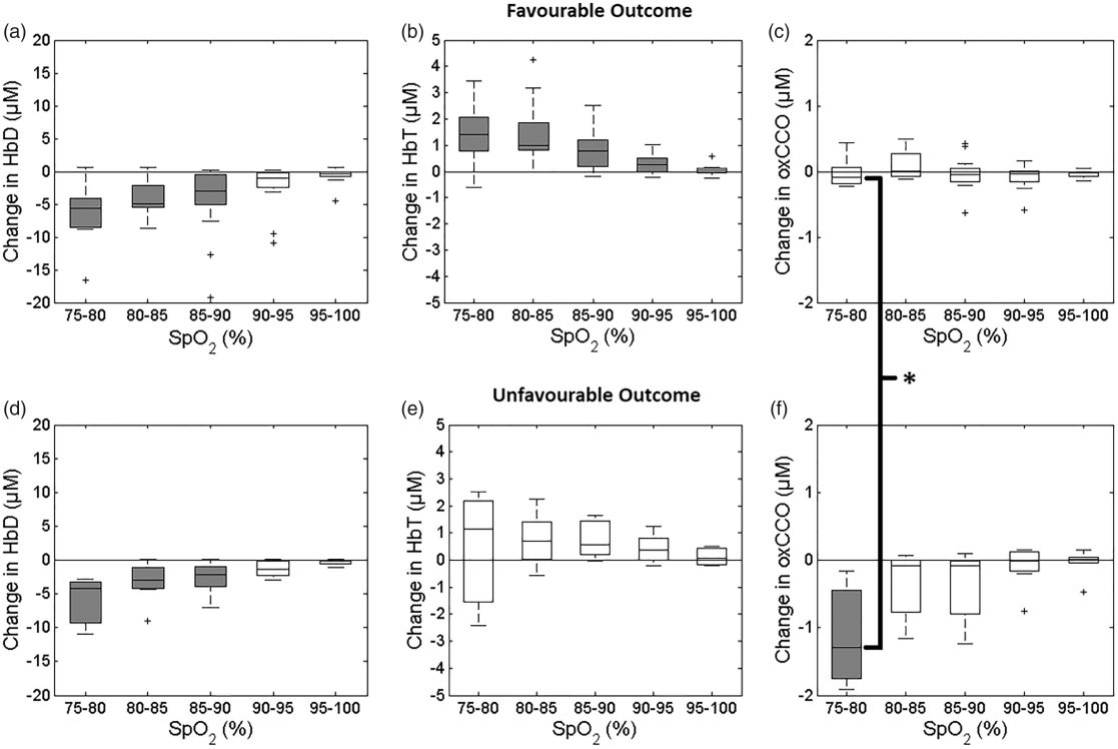
Boxplot showing the change in broadband NIRS measured (a, d) HbD, (b, e) HbT and (c, f) oxCCO with decrease in SpO_2_ during desaturation events in 24 infants: (a–c) 16 favourable outcome, (d–f) 8 unfavourable outcome HIE. Note that the *y*-axis scales are different. The boxplot presents the median and interquartile range, the whiskers show the extreme data points and outliers are presented as crosses. The shaded boxes show a significant difference (*p* < 0.05) from the baseline. The asterisk shows there was a significant difference (*p* = 0.04) in the change in oxCCO between the favourable and unfavourable outcome groups at 75–80% SpO_2_, there was no significant difference in any other variables between the groups.

Three cerebral signals were monitored using broadband NIRS during the desaturation events: cerebral oxygenation, as haemoglobin difference (HbD = HbO_2_−HHb), cerebral blood volume, as total haemoglobin (HbT = HbO_2_ + HHb), and metabolism via oxCCO (see examples in Figure 2(b) and (f)). Figure 3 shows the group changes in each broadband NIRS variable with SpO_2_ during desaturations for the favourable and unfavourable outcome groups. At the lowest SpO_2_ level (75–79%), there was a significantly larger decrease in oxCCO for neonates with unfavourable outcome compared to a favourable outcome (*p* = 0.04) despite no difference in the cerebral oxygenation and blood volume changes (*p* > 0.05). There was no difference between the changes in the haemoglobin signals between the favourable and unfavourable outcome groups. Further, the changes in metabolism from baseline were different between the two groups: there was no change in oxCCO from baseline in the favourable outcome group during desaturation (*p* > 0.05), but there was a large decrease in oxCCO from baseline in the unfavourable outcome group (*p* = 0.04). Across the 4 days, there were no significant temporal relationships observed in the desaturation responses of HbD, HbT or oxCCO, this is true both intra-subject (when there were multiple events) and across the group. In addition, there was no significant relationship of the oxCCO/HbD response with time.

**Figure 4.**
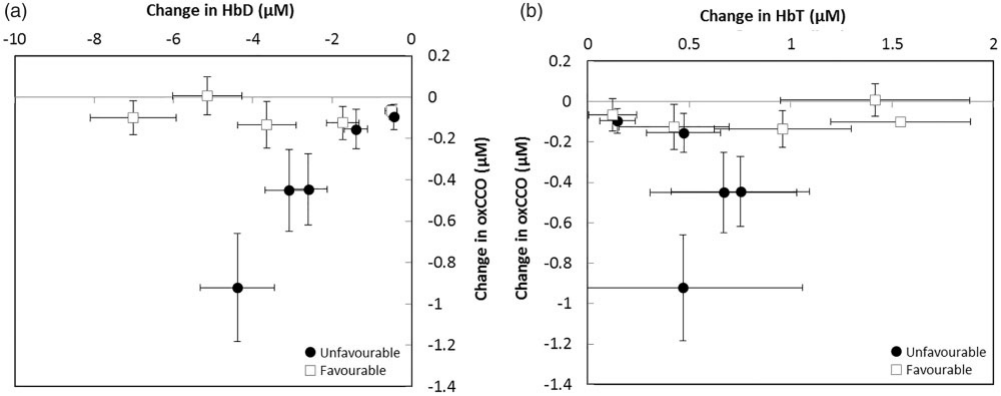
Group cerebral changes during desaturation. (a) Mean change in HbD against mean change in oxCCO per SpO_2_ percentage change for each group. The mean gradients for oxCCO/HbD were −0.004 ± 0.012 (*R*^2^ = 0.032) and 0.21 ± 0.03 (*R*^2^ = 0.93) for the favourable and unfavourable outcome groups, respectively. For patients who experienced multiple desaturation events, the gradients of oxCCO/HbD were consistent across events. For example, patient 021 (favourable outcome) experienced three desaturation events with gradients of −0.008, −0.008 and −0.002 on day 2 (2 events) and 3, respectively, and patient 007 (unfavourable outcome) had 2 events with gradients of 0.18 and 0.14 on day 3. (b) Mean change in HbT against mean change in oxCCO per SpO_2_ percentage change for each group. For oxCCO/HbT the mean gradients were 0.026 ± 0.051 (*R*^2^ = 0.078) and −0.54 ± 0.75 (*R*^2^ = 0.15) for favourable and unfavourable outcome groups, respectively.

**Figure 5.**
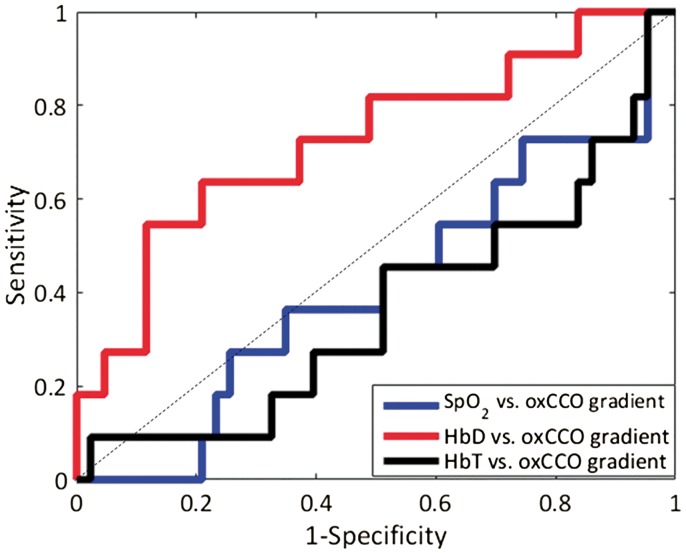
ROC curve for the SpO_2_, HbD and HbT versus oxCCO gradients as biomarkers of outcome. Areas under the curves are 0.41, 0.73 and 0.36 for SpO_2_, HbD and HbT, respectively.

Focussing solely on the cerebral changes occurring during desaturation, Figure 4 shows the group changes in cerebral metabolism with cerebral oxygenation and blood volume, respectively; individual examples are shown in Figure 2(c) and (g). The ROC curves for oxCCO/SpO_2_, oxCCO/HbD and oxCCO/HbT gradients had areas under the curves of 0.41, 0.73 and 0.36, respectively (Figure 5). This showed that oxCCO/HbD gradient was a ‘good’ classifier of outcome: a change in oxCCO of greater than 0.06 µM per 1 µM HbD change indicates unfavourable outcome with 64% sensitivity and 79% specificity.

## Discussion

Cotside assessment of HIE using broadband NIRS can identify differences in the outcome of injury in the first days of life. These data show that the cerebral oxidative metabolism in newborn brain injury behaves differently depending on injury severity; in unfavourable outcome cases of HIE, there is a higher oxygen dependency of mitochondrial metabolism than in favourable outcome cases. This assessment is cotside and provides earlier information than that obtained with MRS on days 5–10; the earliest event recorded was 6 h postpartum.

The HI brain injury severity was not found to impact on the performance of the cerebral haemodynamics or oxygenation. In contrast, the changes in mitochondrial activity during desaturation were related to injury severity. In the favourable outcome group, despite a significant drop in systemic arterial saturation and cerebral tissue oxygenation, there was no change in the metabolic signal (oxCCO). Conversely, a similar oxygenation decrease caused a significant decrease in oxCCO in severely injured neonates (with the unfavourable outcome) indicating a reduction in cerebral metabolism with oxygenation. To put the oxCCO concentration change in perspective, the nadir of the median change in oxCCO was −1.29 µM in the unfavourable outcome group while the total CCO concentration is assumed to be ∼5.5 µM in human brain tissue^28^ (although this is potentially lower in the newborn brain).^38^ Thus, this represents a ∼20% change in the oxidation state of CCO in the brain tissue. These results suggest that cerebral metabolism in severe HI injury is more oxygenation dependent and further, that metabolism becomes oxygen limited at higher oxygenation levels than in HI injury with a favourable outcome. This link between cerebral oxygenation/metabolism and injury implies there is a mismatch between oxygenation and metabolism at the cellular level in HIE with an unfavourable outcome that is not present in cases with a favourable outcome.

The aetiology of the desaturation events is unknown; however, there was no relationship between the number of/absence of/length/depth of the desaturation events and outcome (see Table 1), only the cerebral response to desaturation was injury dependent. The cause of desaturations is unclear as the data was collected during clinically stable periods. The desaturations were not related to seizures as confirmed by continuous EEG and aEEG. In this NICU, the alarm limit is set to SpO_2_ 89–95% and the clinical team responded if (1) SpO_2_ did not spontaneously recover or (2) the desaturation was associated with significant changes in other systemic parameters. Despite these babies being optimally ventilated with indicators of gaseous exchange within normal limits (normal blood gases) and carefully monitored with standard physiological monitoring, these spontaneous episodes of desaturation (mostly self-limiting) were noticed. However, the most interesting outcome of this study was not the occurrence of desaturation events, but rather the cerebral response to these events.

### Mitochondrial metabolism and injury severity

The mechanism behind this effect is likely a decrease in cerebral metabolic rate in more severe brain injury. There is a progressive increase in mitochondrial dysfunction during secondary energy failure,^10^ and therefore, infants with worse outcomes and who are more likely to have experienced secondary energy failure (SEF) will have reduced metabolic capacity. This is supported by MRS studies which have shown that in neonates with severe HIE, there is a larger disturbance of cerebral metabolism than infants with less severe HIE.^39,40^ NIRS studies of neonatal HIE support the hypothesis that HI injury results in a reduced cerebral metabolic rate: Wintermark et al.^20^ observed a lower cerebral metabolic rate of oxygen (CMRO_2_) in moderate compared to severe HI injury; Lemmers et al.^22^ observed a lower cerebral fractional tissue oxygen extraction (FTOE) in infants with adverse outcome; and van Bel et al.^17^ saw a decrease in oxCCO with time from birth in severe HIE (without therapeutic hypothermia), suggesting that the rate of metabolism decreases as the injury progresses. Other studies on this cohort using broadband NIRS have shown a link between metabolic response and injury during systemic changes in HIE,^41,42^ rewarming in HIE,^43^ and stroke.^44^ Further, the response of oxCCO during seizures has been recorded and shown to be unique from the haemodynamic response and potentially related to brain injury.^45^ The mechanism behind this lower metabolic rate in HIE resulting in unfavourable outcome is potentially due to more damage in the mitochondrial respiratory chain from either the initial injury or SEF,^46^ which will result in a lower metabolic rate that is more oxygen-dependent at higher tissue saturation.^47^

Evidence that HIE infants have impaired energy metabolism is also present in other measures of metabolism. HIE results in a reduced cerebral ratio of phosphocreatine to inorganic phosphate (PCr/Pi)^46^ the level of which is predictive of the subsequent neurological outcome.^26,39^ More significantly, lactate is also seen to increase during secondary energy failure^48^ suggesting that impairment in mitochondrial energy metabolism is directly responsible for the secondary fall in PCr and ATP levels. Progressive mitochondrial impairment and impaired oxidative metabolism are thought to be central to the brain lactate increase; the disruption in the balance between cytosolic and mitochondrial ATP-producing metabolic pathways and upregulation of cell membrane transporters such as the sodium-proton exchanger contribute to brain lactate increases acutely.^11^ It has also been shown that there is an increase in apoptosis during SEF which will reduce the available mitochondria.^49^

It is known that therapeutic hypothermia reduces cerebral metabolism.^15,50,51^ This analysis was careful to include only desaturation events occurring at hypothermic temperatures to avoid confounding the analysis; the differences between the injury severities still exist so are additional to, and independent of, the decrease in metabolic rate due to HT. The effect of temperature on oxCCO during rewarming has been analysed separately;^43^ an impaired relationship between cerebral oxygenation and metabolism was related to unfavourable outcome.

Factors that can reduce the activity of CCO have been discussed previously^52,53^ and include: uncoupling of metabolism (when oxygen intake is dependent on the presence of ADP and phosphate) which can be caused by a disruption of the mitochondria;^47^ presence of inhibitors (such as nitric oxide which is known to be associated with HIE); and increased intracellular pH.^26^ A mathematical model of physiology found that mitochondrial uncoupling and the death of brain tissue are the most important factors in understanding the decrease in metabolism after severe HIE.^54^ Interestingly, the extent of brain injury has been shown to be associated with an alkaline intracellular pH;^55^ therefore, a likely hypothesis is that increased pH is a factor in the observed increased dependence of oxCCO on oxygenation in HIE resulting in unfavourable outcome.

### Oxygen dependency of mitochondrial metabolism indicates injury severity

The reduced mitochondrial function can alter cellular oxygen dependency. A reduction in arterial oxygenation will decrease oxygen delivery (as observed in the reduction of HbD during the desaturations) eventually decreasing oxidative metabolism once the tissue partial pressure of oxygen has decreased. Mitochondria in mild to moderate brain injury have a lower oxygen saturation threshold or ‘critical mitochondrial oxygenation’; in less severe brain injury, the oxygen tension in the majority of mitochondria is above the value at which their redox state becomes oxygen dependent, and it is not until there is a substantial fall in oxygen tension that a sufficiently large population of mitochondria have an oxygen tension low enough to affect the measured CCO oxidation state.^30^ This might be because impaired mitochondria require a higher tissue oxygenation to function, or because an increased oxygen tension gradient is required to drive oxygen across oedematous tissue to reach the mitochondria.^32^ Banaji et al.^53^ predicted how the oxCCO signal should respond during an oxygen desaturation using a mathematical model of brain circulation and energy metabolism: during hypoxia in a healthy brain there is an approximately linear relationship with HbO_2_; when CMRO_2_ is lowered by 60%, however, the relationship becomes biphasic and much larger changes in the oxCCO signal can occur for similar saturation changes. In HIE with an unfavourable outcome, we observed the oxygenation threshold of metabolism is reached at a higher oxygenation level than in HIE with a favourable outcome.

A biphasic oxCCO change has also been seen in animal models of HI.^30,56,57^ Bainbridge et al.^56^ interpreted the decrease in oxCCO as an impairment of the ETC, which in turn results in failure of ATP production through ATP synthase.^56^ The threshold point in the double-linear fit of the data has been interpreted as the point at which ATP manufacture in oxidative metabolism is almost completely suppressed. Springett et al.^30^ observed a biphasic oxCCO response during anoxia in piglets, further demonstrating a shift in the threshold if the animal is hypercapnic.^30^ The authors postulate that the point at which oxCCO suddenly decreases is caused when the ETC becomes oxygen limited. Cooper et al.^57^ also reported a biphasic relationship between metabolism and perfusion (via CMRO_2_ and CBF) in a piglet artery occlusion model measured using NIRS. Additionally, hyperoxia in acute brain injury patients suggested a change in mitochondrial redox status and the presence of oxygen-dependent metabolism above traditionally described ischaemic thresholds.^58^

Healthy adult studies of hypoxemia have not observed a biphasic oxCCO relationship with changes in oxygen saturation.^33^ One study reported a linear correlation between oxCCO and cerebral oxygen delivery during the oxygen desaturations but did not find a relationship between oxCCO and HbD.^59^ It is possible that the lack of threshold observed is because the saturation cannot be safely and ethically brought low enough to reach CCO reduction in healthy adult volunteers.

Further evidence that the coupling between oxygenation and metabolism is related to metabolic rate is presented by Wilson et al.^47^ The authors found that at high oxygen tensions, the redox state of CCO is independent of oxygen tension and determined by the metabolic state and the activity of the tricarboxylic acid cycle. As oxygen tension reduced to zero, all the components of the ETC became reduced, and the point at which oxidation changes are first observed in CCO depends on the metabolic state. The measured dependences of the rate of mitochondrial metabolism and of the reduction of the ETC components on oxygen concentration are not constant but change dramatically with changes in metabolic status.

### Limitations and future directions

The next step for this work is to evaluate the prognostic utility of the oxCCO signal in the first hours after injury; a central goal of cotside monitoring is to identify high-risk infants in the first 6 h after injury when further interventions or adjunct therapies might be beneficial. Due to difficulties with obtaining consent, it was very difficult to monitor every neonate as early as 6 h postpartum (it was only possible in one case, neonate 60, where we were able to begin monitoring at 5 h postpartum) but with the promise of broadband NIRS shown in this study and others,^42^ it is now essential that we investigate the signal as an early biomarker of tissue health. A further goal would be to monitor the progression of tissue metabolism from the first hours of injury, throughout hypothermia and re-warming to assess tissue health throughout treatment. This would require continuous monitoring but would give insight into the mitochondrial injury, metabolic dysfunction and cell death associated with SEF in real-time.

Combining the broadband NIRS oxCCO signal with other cotside monitors of metabolism, such as FTOE^60^ or CMRO_2_^15^ would give additional information regarding the initial metabolic status of the brain before the desaturation events and would confirm our hypothesis that the more severely injured brain has a lower metabolic rate. The instrument presented here is customisable and can be upgraded to measure FTOE via tissue saturation measured by spatially resolved spectroscopy^61^ or broadband spectrum fitting.^62^

Finally, as many neonates with HIE experience seizures and abnormal electrical activity, it is interesting to assess the relationship between oxCCO and EEG. This has been done in a case study of an infant with multiple seizure events,^45^ and it is being investigated further in seizures and during clinically stable periods. We know that there was no seizure activity during the desaturations events analysed here, but it will be interesting to see if there are any changes in electrical activity associated with the events, or the metabolic response to electrical activity.

### Conclusion

In conclusion, we have shown that the cerebral oxidative metabolism in newborn brain injury behaves differently depending on injury severity. In particular, we have shown that in HIE with unfavourable outcome there is a higher mitochondrial dependence on oxygenation. We postulate that this is due to mitochondrial dysfunction and reduction in cerebral metabolic rate as a result of severe encephalopathy. In addition, uniquely we have obtained these results using a non-invasive bedside technique, broadband NIRS, in the first 4 days of life, which is earlier than it is possible to perform MRS, the gold standard marker of outcome. While further work on the implementation and applicability of the broadband NIRS technique is needed, the combined measurement of changes in haemoglobin oxygenation and CCO oxidation have the potential to provide a non-invasive and cotside marker of neurodevelopmental outcome following neonatal HI injury.

## Supplemental Material

Supplemental material for Oxygen dependency of mitochondrial metabolism indicates outcome of newborn brain injury Click here for additional data file. Supplemental material for Oxygen dependency of mitochondrial metabolism indicates outcome of newborn brain injury by Gemma Bale, Subhabrata Mitra, Isabel de Roever, Magdalena Sokolska, David Price, Alan Bainbridge, Roxana Gunny, Cristina Uria-Avellanal, Giles S Kendall, Judith Meek, Nicola J Robertson and Ilias Tachtsidis in Journal of Cerebral Blood Flow & Metabolism
