# Supplementary material for: Oxygen dependency of mitochondrial metabolism indicates outcome of newborn brain injury
Source: J Cereb Blood Flow Metab. 2018 May 18;39(10):2035–47. doi: 10.1177/0271678X18777928 (PMC6775592; doi:10.1177/0271678X18777928)
Supplement: Supplemental material for Oxygen dependency of mitochondrial metabolism indicates outcome of newborn brain injury [file Supplemental_material_10.pdf]

Supplementary Table 1: Details of all neonates studied including those without desaturation events eligible for analysis.

Neonate	Gender	GA (weeks)	Birth Weight (g)	Lac/NAA	Outcome Prediction	Duration (hours)	No. of events
2	F	38.0	1770	0.87	Unfavourable	29.0	1
3	F	41.0	3800	0.2	Favourable	47.1	9
4	M	40.1	3310	0.16	Favourable	31.7	0
6	M	40.3	3110	0.39	Unfavourable	29.1	2
7	M	39.7	3640	1.32	Unfavourable	24.7	2
8	F	41.9	3498	0.17	Favourable	26.8	1
9	M	38.9	2850	0.16	Favourable	35.3	5
10	F	40.3	3020	0.15	Favourable	61.8	0
11	F	40.6	3580	0.08	Favourable	77.5	1
12	M	39.0	2250	1.8	Unfavourable	22.5	0
13	M	39.1	3060	0.76	Unfavourable	77.5	0
14	M	37.7	3750	0.25	Favourable	30.9	2
15	F	39.6	3240	0.15	Favourable	36.4	3
16	M	40.0	3770	Died	Unfavourable	24.2	0
17	F	37.4	3160	0.35	Unfavourable	50.2	2
18	F	38.3	3034	0.28	Favourable	49.5	0
19	F	39.4	3700	0.16	Favourable	28.9	1
20	M	40.9	3190	0.11	Favourable	48.7	2
21	M	36.4	2440	0.2	Favourable	41.1	3
22	M	41.6	3910	0.2	Favourable	27.4	0
25	M	41.9	4940	0.4	Unfavourable	8.1	1
26	M	39.4	2811	0.52	Unfavourable	32.7	0
28	M	39.9	3200	0.16	Favourable	4.1	0
29	M	42.1	4280	No data	No data	3.0	0
30	M	40.0	3000	0.17	Favourable	32.5	0
31	M	41.1	3145	1	Unfavourable	33.9	0
33	F	38.4	2900	0.43	Unfavourable	47.2	0
35	F	38.7	3150	0.14	Favourable	12.0	1
37	F	39.4	3330	0.2	Favourable	10.1	2
39	M	37.1	3334	0.18	Favourable	46.3	0

40	F	41.0	3050	0.14	Favourable	41.9	0
42	F	37.9	2350	0.23	Favourable	6.1	0
44	M	40.0	3060	No data	No data	49.8	0
45	M	37.1	2510	0.2	Favourable	45.0	0
47	F	41.7	3390	0.41	Unfavourable	16.9	1
48	M	39.0	3340	2.64	Unfavourable	29.0	1
49	F	35.3	2250	0.22	Favourable	49.0	0
50	M	39.4	3460	0.91	Unfavourable	62.2	0
51	M	38.1	3070	0.18	Favourable	15.6	2
53	F	37.6	3020	0.17	Favourable	16.9	2
54	F	38.3	3490	0.19	Favourable	58.5	0
55	F	40.3	4260	0.19	Favourable	18.7	0
56	M	40.0	3365	0.34	Unfavourable	13.5	1
57	M	39.6	3370	0.15	Favourable	1.5	0
58	M	41.9	3830	0.16	Favourable	8.1	0
59	M	41.1	3990	0.16	Favourable	15.5	3
60	F	40.7	2754	0.15	Favourable	15.7	3
61	F	41.4	4379	0.13	Favourable	6.1	0
62	F	40.0	3442	0.21	Favourable	25.8	3
63	F	39.6	1790	0.54	Favourable	12.6	0
